# Medical Students’ Opinion of Their Learning Process

**DOI:** 10.1007/s40670-023-01873-1

**Published:** 2023-09-06

**Authors:** Eva Montané, Cristina Vilaplana, Joan Riera, Marina Pujol, Maria Méndez, Albert Mas, Angel Vara, David Parés

**Affiliations:** 1 Department of Pharmacology, Therapeutics and Toxicology, Universitat Autònoma de Barcelona, Bellaterra, Spain; 2 Department of Clinical Pharmacology, Hospital Universitari Germans Trias i Pujol, Carretera de Canyet s/n, 08916 Badalona, Barcelona, Spain; 3 Experimental TB Unit, Department of Microbiology, Northern Metropolitan Clinical Laboratory, Hospital Universitari Germans Trias i Pujol, Badalona, Barcelona, Spain; 4 Infectious Diseases and International Health Clinical Division, Northern Metropolitan Management of the Catalan Institute of Health, Badalona, Spain; 5 Medical School at Hospital Universitari Germans Trias i Pujol, Badalona, Barcelona, Spain, Universitat Autònoma de Barcelona, Bellaterra, Spain; 6 Department of Paediatrics, Hospital Universitari Germans Trias i Pujol, Badalona, Barcelona, Spain; 7 Department of Paediatrics, Obstetrics and Gynecology, Preventive Medicine and Public Health, Universitat Autònoma de Barcelona, Bellaterra, Spain; 8 Department of Surgery, Hospital Universitari Germans Trias i Pujol, Badalona, Barcelona, Spain; 9 Department of Surgery, Universitat Autònoma de Barcelona, Bellaterra, Spain

**Keywords:** Academic research, Education graduate students, Learning process, Opinion, School of medicine, Undergraduate medical curriculum, Web-based questionnaire

## Abstract

**Introduction:**

The opinion of students is of utmost importance to identify areas of improvement in undergraduate studies. Medical schools would use this information to plan actions to ensure that the students achieve the necessary medical knowledge. The aim of this study was to analyse the opinion of medical students about their learning process and to analyse the influence of their experience according to their year of medical degree.

**Methods:**

A questionnaire including 21 items, divided into four sections (motivation, theory lectures, hospital internships, and research) and two overall questions, was distributed among eligible 246 students. Each item was scored from 1 (strongly disagree) to 5 (strongly agree). The opinions of intermediate-year students of medical degree (3rd and 4th) were compared to late-year students (5th and 6th).

**Results:**

A total of 148 students answered the questionnaire (60.2% response rate). The mean scores for overall student motivation and teaching quality were 6.15 and 7.10, respectively. The student–teacher interaction and new learning technological tools were considered important for student motivation. The only differences found between the two groups of students were that late-year students wished to become part of a medical team and to learn writing scientific papers more than the intermediate-year students.

**Conclusions:**

This questionnaire revealed that the year of career had little influence on the medical students’ opinion on their learning process during their undergraduate studies. Late-year students rated highest on being more interested in being part of a medical team and their knowledge on writing scientific articles. The use of new technologies and the student–teacher interaction is key to motivate students.

## Introduction

Medicine studies are from ancient times the paradigm of university, where teaching not only aims to acquire new theoretical knowledge, but also develop technical skills. Therefore, to design, to plan, and to develop an adapted learning environment engaged with medical curricula is of paramount importance [1].

With recent world changes, university undergraduate teaching programs have to be linked with new expectations. Therefore, new methods for knowledge transfer, new technology tools and advances on design thinking, changed the classical structure of undergraduate studies, especially in medical studies [2–4].

Recently, a change of motivation pattern among youth and, consequently, among university students has been demonstrated. Also, burnout and changes on how to engage with students play a role in the landscape of undergraduate studies redesign [5, 6].

Motivation is essential for engaging students in their learning process being a predictor of their academic performance [7–9]. This motivation could change depending on their year of medical degree, from the beginning to the end of the career, as many factors that are involved in motivation such as autonomy, competence, and the relationship changes during the degree [10]. Thus, the opinion of students is of utmost importance to identify areas of improvement in undergraduate studies. Medical schools would use students’ motivation information, to plan changes and to introduce an action plan to assure they achieve medical knowledge. In fact, motivation related to methods of learning is clearly linked to student’s perceptions and the achievement of good learning outcomes [11].

To date, several surveys have been conducted exploring the students’ opinion on specific tools or learning skills such as problem-based learning [12] or assessing the opinion on specific learning subjects such as psychiatry [13] or statistics [14], or focusing on specific implemented learning modules such as scientific terms in the Faculty of Medicine Charite [15]. To our knowledge, data on the influence depending on the year of career in the medical students’ opinion about their learning process is scarce.

The aim of this study was to analyse the opinion of medical students on the quality and characteristics of their learning process, in order to introduce structured changes that could improve their learning process, focusing on whether there was any difference according to the year of medical degree of each responder.

## Materials and Methods

### Study Design

This study analyses the opinions of medical students in their third to sixth year of the Bachelor of Medicine program regarding their learning process. We conducted a questionnaire specially designed for this study in Spanish.

### Setting

The medical degree in Spain is a 6-year university course of study. This study was carried out at the Faculty of Medicine of the Germans Trias i Pujol University Hospital (Barcelona, Catalonia, Spain), which receives students from the third to the sixth year of medical school. In the clinical setting, the vast majority of teachers are physicians who perform clinical activities in hospital or outpatient care. The main teaching modalities used in our School of Medicine are theoretical classes, clinical seminars, laboratory practices, hospital internships, and simulation practices. The initial years of the degree, first and second, are considered foundational for the learning of basic medical sciences, and they are taken in a different university campus with a majority of teachers who do not carry out clinical activities.

### Study Population

All students of our School of Medicine were invited to fill in the questionnaire. In the 2017–2018 academic years, a total of 246 students were enrolled in the program.

In order to analyse the influence of the experience as student based on their year of career, students who answered the questionnaire were divided into two groups: medical students in intermediate years (students who were in their third or fourth year) and medical students in late years (for those that were in the fifth or sixth year).

### Outcome Measures

The anonymous questionnaire was developed ad hoc by four teachers (ME, VC, MM, PD) and four students (RJ, PM, AM, VA). When the preliminary version of the questionnaire was written, it was first distributed to a workgroup of 54 teachers and students to discuss changes to improve it and a final version of the questionnaire was created.

The questionnaire was performed using the Typeform® software (Typeform SL, Barcelona, Spain) and complied with the European General Data Protection Regulation. At the beginning of the questionnaire, there was an introduction explaining its objectives, as well as encouraging responses. It also mentioned that the responses would be anonymous and voluntary. The web-based questionnaire took approximately 10 min to complete and it could be filled through a computer, a tablet, or a smartphone.

In July 2018, the questionnaire was shared through the university alumni mailing list and the university administrative staff sent two reminders, each one a week apart, in order to increase the answer rate.

The questionnaire included 40 closed questions distributed in four sections or fields: motivation, theory lectures, hospital internships, and medical research. For most of the questions, participants were asked to rank their agreement with the questions through a Likert scale from 1 to 5, where 1 meant to strongly disagree with the topic; 2 disagree; 3 neither agree nor disagree; 4 agree; and 5 strongly agree. There were also two overall questions about motivation and teaching quality, rating from 1 to 10, and two open questions per section, designed to collect detailed information on the medical students’ views, worries, and improvement proposals.

In this article, we have done a selection of the questions that we are presenting. We have excluded those that asked for local aspects that could not be of real interest to other universities, resulting in a questionnaire with 21 closed questions distributed as: two overall questions, five questions on motivation, seven on theory lectures, three on hospital internship, and four on medical research (Table 1). The questions not included in this work were related to the notes of the theoretical courses provided by teachers or elaborated by the students; since in our university, medical students are organized to collect and summarize the content of the theoretical classes they attend to share with the rest of their classmates. In addition, we have excluded questions related to a competitive examination taken by doctors in Spain to access a specialty called “Médico Interno Residente” exam (or MIR exam).

**Table 1:** Description of the questionnaire

**Fields of questions**		**Questions**	**Range score (points)**
Motivation (M)	Please rate the level of agreement on the following actions for student motivation:		
	M1	Assisting theory lectures	1–5
	M2	Encouraging student–teacher interaction	1–5
	M3	Offering complementary activities to improve grades	1–5
	M4	Incorporating new technology tools during lectures	1–5
	M5	Seeking feedback on subjects	1–5
Theory lectures (TL)	Please rate the level of agreement on the importance of the following statements about theory lectures:		
	TL1	They are useful for learning	1–5
	TL2	They are useful for passing a subject	1–5
	TL3	Encouraging students to read materials in advance	1–5
	TL4	Facilitating active participation	1–5
	TL5	Providing key highlights of important concepts	1–5
	TL6	Utilizing case reports for better conceptual understanding	1–5
	TL7	Limiting lesson duration to a maximum of 50 min	1–5
Hospital internships (HI)	Please rate the level of agreement on the importance of the following statements about hospital internship:		
	HI1	They areuseful for acquiring skills and knowledge	1–5
	HI2	Integrating students into medical teams	1–5
	HI3	Establishing clear objectives and activities for training in advance	1–5
Research (R)	Please rate the level of agreement on the importance of the following statements about research:		
	R1	Research is important in the academic development of medical students	1–5
	R2	Courses should incorporate research components	1–5
	R3	Offering optional participation in a research group is beneficial	1–5
	R4	Teaching medical students how to write scientific papers and abstracts for conferences	1–5
Overall (O)	Please rate the level of the following:		
	O1	Overall students’ motivation	0–10
	O2	Overall quality of teaching	0–10

### Statistical Analysis

A descriptive analysis was performed. The answer rate of the questionnaire was calculated by dividing the number of responses by the total number of students matriculated. For each question of the questionnaire, the mean score (SD) and the score range were provided for the two scales used (5-points Likert scale or 10-points overall scale). For the comparison analysis between the year of career (intermediate vs. late years), the *t* Student test was used for every mean score question. A bilateral *p*-value < 0.05 was used to determine statistical significance.

Statistical analysis was performed using the SPSS statistical software package for Windows, version 15.0 (SPSS™ Inc., Chicago, IL, USA).

### Ethical Considerations

Before starting the questionnaire, participants were informed about the aim of the study and the compliance with their rights. By answering the questionnaire, the students agreed on participating in the questionnaire. Data was collected and analysed anonymously.

## Results

### Sample

An overall of 148 answers have been obtained out of 246 matriculate students (60.2% response rate). The questionnaire was answered from a smartphone in 88.5% of the answers (131), from a computer or laptop in 10.8% (16), and from a tablet in 0.7% (1). The average response time of the questionnaire was 11 min and 8 s.

The year of medical degree with a higher response rate was the fifth year (52/61, 85.2%), and the one with the lower response rate was the sixth year (20/47, 42.6%). The response rate was higher in the late-year students compared to the intermediate-year students (66.7% vs. 55.1%) (Table 2).

**Table 2:** Answer rates according to the year career course of medical students

**Year career course of medical students**	**Third**	**Fourth**	**Fifth**	**Sixth**	**Total** ***N*** **(%)**
	**Intermediate-year** ***N* (%)**		**Late-year** ***N* (%)**		
**Matriculate students** ***N* (%)**	72 (29.3)	66 (26.8)	61 (24.8)	47 (19.1)	246 (100)
	138 (56.1)		108 (43.9)		
**Answers** ***N* (%)**	33 (45.8)	43 (65.1)	52 (85.2)	20 (42.6)	148 (60.2)
	76 (55.1)		72 (66.7)		

All the questionnaire results are detailed in Table 3 and Table 4. Table 3 shows the mean (SD) scores and score ranges of each question from the total responses, and Table 4 shows the mean (SD) scores and score ranges of each question for each of the student group (intermediate year and late year) and their statistical comparison.

**Table 3:** Mean scores (SD) and range for each question

Question		Mean (SD) score	Range score
Motivation (M): Please rate the level of agreement on the following actions for student motivation:			
M1	Assisting theory lectures	2.1 (0.84)	1–4
M2	Encouraging student–teacher interaction	3.9 (1.13)	1–5
M3	Offering complementary activities to improve grades	3.5 (1.12)	1–5
M4	Incorporating new technology tools during lectures	4.3 (0.85)	1–5
M5	Seeking feedback on subjects	4.3 (0.93)	1–5
Theory lectures (TL): Please rate the level of agreement on the importance of the following statements about theory lectures:			
TL1	They are useful for learning	3.53 (1.03)	1–5
TL2	They are useful for passing a subject	3.20 (1.18)	1–5
TL3	Encouraging students to read materials in advance	3.18 (1.09)	1–5
TL4	Facilitating active participation	4.15 (0.81)	1–5
TL5	Providing key highlights of important concepts	4.80 (0.45)	2–5
TL6	Utilizing case reports for better conceptual understanding	4.60 (0.68)	2–5
TL7	Limiting lesson duration to a maximum of 50 min	4.50 (0.90)	1–5
Hospital internships (HI): Please rate the level of agreement on the importance of the following statements about hospital internships:			
HI1	They are useful for acquiring skills and knowledge	4.17 (1.03)	1–5
HI2	Integrating students into medical teams	4.50 (0.72)	2–5
HI3	Establishing clear objectives and activities for training in advance	4.41 (0.83)	1–5
Research (R): Please rate the level of agreement on the importance of the following statements about research:			
R1	Research is important in the academic development of medical students	3.88 (0.92)	1–5
R2	Courses should incorporate research components	3.63 (1.02)	1–5
R3	Offering optional participation in a research group is beneficial	4.20 (0.99)	1–5
R4	Teaching medical students how to write scientific papers and abstracts for conferences	4.24 (0.92)	1–5
Overall (O): Please rate the level of the following:			
O1	Overall students’ motivation	6.15 (1.6)	1–9
O2	Overall quality of teaching	7.10 (1.4)	1–10

**Table 4:** Comparison of mean scores of each question between groups of medical students

Question		Intermediate-year students (*n* = 76)	Late-year students (*n* = 72)	*p*
Motivation (M): Please rate the level of agreement on the following actions for student motivation:				
M1	Assisting theory lectures	2.1 (0.8)	2.0 (0.8)	0.515
M2	Encouraging student–teacher interaction	3.7 (1.2)	4.1 (1.1)	0.073
M3	Offering complementary activities to improve grades	3.3 (1.1)	3.6 (1.1)	0.123
M4	Incorporating new technology tools during lectures	4.3 (0.8)	4.2 (0.9)	0.707
M5	Seeking feedback on subjects	4.1 (1.0)	4.4 (0.8)	0.110
Theory lectures (TL): Please rate the level of agreement on the importance of the following statements about theory lectures				
TL1	They are useful for learning	3.41 (1.05)	3.65 (0.99)	0.148
TL2	They are beneficial for passing a subject	3.10 (1.25)	3.30 (1.11)	0.339
TL3	Encouraging students to read materials in advance	3.16 (1.10)	3.20 (1.10)	0.840
TL4	Facilitating active participation	4.05 (0.92)	4.26 (0.67)	0.115
TL5	Providing key highlights of important concepts	4.83 (0.53)	4.86 (0.35)	0.664
TL6	Utilizing case reports for better conceptual understanding	4.51 (0.77)	4.68 (0.65)	0.134
TL7	Limiting lesson duration to a maximum of 50 min	4.49 (0.99)	4.56 (0.80)	0.644
Hospital internships (HI): Please rate the level of agreement on the importance of the following statements about hospital internships				
HI1	They are useful for acquiring skills and knowledge	4.04 (1.14)	4.31 (0.88)	0.115
HI2	Integrating students into medical teams	4.34 (0.81)	4.65 (0.58)	0.009
HI3	Establishing clear objectives and activities for training in advance	4.39 (0.80)	4.43 (0.87)	0.795
Research: Please rate the level of agreement on the importance of the following statements about research				
R1	Research is important in the academic development of medical students	3.88 (0.98)	3.87 (0.85)	0.965
R2	Courses should incorporate research components	3.54 (1.10)	3.74 (0.93)	0.245
R3	Offering optional participation in a research group is beneficial	4.22 (0.92)	4.18 (1.07)	0.792
R4	Teaching medical students how to write scientific papers and abstracts for conferences	4.09 (0.99)	4.40 (0.80)	0.039
Overall (O): Please rate the level of the following				
O1	Overall students’ motivation	6.26 (1.66)	6.04 (1.51)	0.399
O2	Overall quality of teaching	7.08 (1.32)	7.11 (1.41)	0.886

### Questions About Overall Motivation and Quality of Teaching (O)

The mean (SD) score of the overall students’ motivation (item O1) was 6.15 (1.6) ranging from 1 to 9. The mean (SD) score of the overall perception of quality of teaching (item O2) was 7.10 (1.4) ranging from 1 to 10 (Table 3). No statistical differences were found between the mean scores of those answers comparing students of two groups (*p* = 0.399 and *p* = 0.886, respectively) (Table 4).

### Questions About the Students’ Motivation (M)

The mean scores of all questions related to motivation are detailed in Table 3. To assist theory lectures (item M1) did not motivate the student (mean 2.1); and contrarily, the student–teacher interaction (seminars) (item M2), new technology tools used during courses (item M4), and giving feedback about any medical topic (discussion lectures) (item M5) motivated the students (the mean of M2 question was 3.9 and the mean of M4 and M5 questions was 4.3). There were no statistical differences when comparing all motivation questions among groups (Table 4).

### Questions About Theory Lectures (TL)

The mean scores of all questions related to theory lectures are detailed in Table 3. The teacher’s emphasis on relevant concepts (item TL5) and the use of case reports to complete understandable concepts (item TL6) were both important for students (mean 4.8 and 4.6, respectively). When comparing theory lectures questions among groups, no statistical differences were found (Table 4).

### Questions About Hospital Internships (HI)

The mean scores of all questions related to hospital internships are detailed in Table 3. When we compared all questions between intermediate-year and late-year students, no statistical differences were found. The only difference observed was in the question related to the importance to become part of the medical team (item HI2), where late-year students had a higher score (4.3 vs. 4.6; *p* = 0.009) (Table 4).

### Questions About Research (R)

The mean scores of all questions related to medical research and innovation are detailed in Table 3. There were no statistical differences between both groups except for the question about the wish to learn how to write medical scientific articles and abstracts for conferences (item R4), where late-year students had a higher score (4.1 vs. 4.4; *p* = 0.039) (Table 4).

## Discussion

Motivation is key for engaging students in the medical learning process. This motivation could be different depending on the year of career of the student. Therefore, the opinion of students is crucial to identify areas of improvement in undergraduate studies. In the present study, we aimed to analyse the opinion of medical students regarding quality and characteristics of their learning process in order to implement structured changes that could improve the learning process. We found interesting results, particularly concerning various aspects of teaching, including lectures, hospital internships, and medical research.

Results regarding questionnaire studies are more accurate depending on the response rate. A 60% of students answered the questionnaire in our study, and therefore it could be considered that is a high response rate. This can be attributed to the fact that when the topics of the questionnaire are relevant and meaningful to the participants, it is more likely to impact their response rates [16]. In our study, some students were very involved from the beginning, both in the design and performance of the project, and in the analysis of its results. Therefore, there is a relationship of experience as a medical student and rate of response as late-year students had a higher response rate. Overall, students considered they had a medium–high motivation; and they considered the quality of teaching received as a high; however, as recent reports support this belief [2] and has been perceived among university students, measures to enhance the quality of education should be considered to further improve the learning process.

“Assisting theory lectures to motivate students” (item M1) was the item with which all students agreed the least (lowest score), which explains the wide lack of class attendance. As it was expected, students expressed the need for the introduction of new learning methods (including simulation, flipped classes, additional seminars for cooperative learning, discussion, and clinical cases), increasing the student participation in the learning process and with the use of new technologies. This is presented by Pickering and Swinnerton and Ekstrand et al. as a good attitude trigger [7, 17]. In addition, they wanted to be part of medical teams during hospital internship and to be trained in medical research [18]. Probably, as it was stated, this part of medical professionalism can be introduced with a structured mentorship program [6].

The information contained in our results pointed out the importance of the benefits of introduction of innovation in learning processes in the next future as well as the effects of engaging in medical teams during hospital internships. In addition, especially the late-year students expressed the viewpoint that gaining practical knowledge in research activities, including scientific article writing and participation in medical conferences, is of great importance. In our opinion, this would be an endpoint for redesigning medical studies. The active participation, through co-creation, of medical students in medical studies curricula is then warranted [18].

This study is based on answers from a questionnaire. Although the response rate was high, there are some limitations. Firstly, there is a selection bias, since the most motivated students are generally who answer the questions. In addition, late-year students have more experience and have a broader opinion on how the career should be. Furthermore, it is important to acknowledge that this questionnaire was distributed to a single medical school, which may limit its representativeness for students from other universities. Another potential limitation is that the questionnaire was administered in 2018, before the emergence of the SARS-CoV-2 pandemic, which might have changed students’ opinions following changes made to teaching programs [19].

The main strength of our study is that this is the first study exploring the opinions of medical students regarding their learning process through years of graduation, providing relevant information that could be useful to other medical schools. In addition, more than fifty students and teachers participated in the design of the questionnaire, which demonstrates the high degree of involvement in this project. Our results have been used in our centre as a basis for several actions taken to improve the teaching and learning process, such as increasing simulation-based learning, changing the schedule of hospital internship to make it easier for students to be a part of medical team, as well as increasing the number of flipped classes to promote teacher-student interaction. Moreover, a recent novelty is that certain students have the opportunity to carry out medical research internships.

In conclusion, the year of career had little influence on the medical students’ opinion on their learning process during their undergraduate studies. However, we found a significant motivation among medical students to implement changes in order to improve their learning process. They asked to introduce innovative teaching methodologies to modernize the process of learning in medical studies. Medical research would also be a desirable milestone to be introduced in medical school curricula.

## Data Availability

The datasets used and/or analysed during the current study are available from the corresponding author on reasonable request.

## References

[1] Karani R (2015). Enhancing the medical school learning environment: a complex challenge. J Gen Intern Med.

[2] Wouters A, Croiset G, Schripsema NR, Cohen-Schotanus J, Spaai GWG, Hulsman RL, Kusurkar RA (2017). Students’ approaches to medical school choice: relationship with students’ characteristics and motivation. Int J Med Educ.

[3] Jung H, An J, Park KH (2018). Analysis of satisfaction and academic achievement of medical students in a flipped class. Korean J Med Educ.

[4] Spencer J, Blackmore D, Heard S, McCrorie P, McHaffie D, Scherpbier A, Gupta TS, Singh K, Southgate L (2000). Patient-oriented learning: a review of the role of the patient in the education of medical students. Med Educ.

[5] Boni RA dos S, Paiva CE, de Oliveira MA, Lucchetti G, Fregnani, JHTG, Paiva BSR. Burnout among medical students during the first years of undergraduate school: prevalence and associated factors. PLoS One. 2018. 10.1371/journal.pone.0191746. 10.1371/journal.pone.0191746 PMC5841647 29513668

[6] Dussán KB, Leidal A, Corriveau N, Montgomery D, Eagle KA, LaHood BJ (2017). Increasing medical trainees’ empathy through volunteerism and mentorship. J Med Educ Curric Dev.

[7] Pickering JD, Swinnerton BJ (2018). Exploring the dimensions of medical student engagement with technology-enhanced learning resources and assessing the impact on assessment outcomes. Anat Sci Educ.

[8] Moxham BJ, Plaisant O (2007). Perception of medical students towards the clinical relevance of anatomy. Clin Anat.

[9] Kerby J, Shukur ZN, Shalhoub J (2011). The relationships between learning outcomes and methods of teaching anatomy as perceived by medical students. Clin Anat.

[10] Steinmayr R, Weidinger AF, Schwinger M, Spinath B (2019). The importance of students’ motivation for their academic achievement - replicating and extending previous findings. Front Psychol.

[11] Kusurkar RA, Ten Cate TJ, van Asperen M, Croiset G (2011). Motivation as an independent and a dependent variable in medical education: a review of the literature. Med Teach.

[12] Musal B, Gursel Y, Taskiran HC, Ozan S, Tuna A (2004). Perceptions of first and third year medical students on self-study and reporting processes of problem-based learning. BMC Med Educ.

[13] Chew QH, Tan E, Sum MY, Sim K (2021). Inter-relationships between perception of educational environment and learning processes within medical undergraduate psychiatry teaching: a mediational analysis. Med Educ Online.

[14] Zhang Y, Shang L, Wang R, Zhao Q, Li C, Xu Y, Su H (2012). Attitudes toward statistics in medical postgraduates: measuring, evaluating and monitoring. BMC Med Educ.

[15] Drees S, Schmitzberger F, Grohmann G, Peters H (2019). The scientific term paper at the Charité: a project report on concept, implementation, and students’ evaluation and learning. GMS J Med Educ.

[16] Fan W, Yan Z (2010). Factors affecting response rate of the web survey: a systematic review. Comput Human Behav.

[17] Ekstrand C, Jamal A, Nguyen R, Kudryk A, Mann J, Mendez I (2018). Immersive and interactive virtual reality to improve learning and retention of neuroanatomy in medical students: a randomized controlled study. CMAJ Open.

[18] Ratte A, Drees S, Schmidt-Ott T (2018). The importance of scientific competencies in German medical curricula - the student perspective. BMC Med Educ.

[19] Lee IR, Kim HW, Lee Y, Koyanagi A, Jacob L, An S, Shin JI, Smith L. Changes in undergraduate medical education due to COVID-19: a systematic review. Eur Rev Med Pharmacol Sci. 2021. 10.26355/eurrev_202106_26155. 10.26355/eurrev_202106_26155 34227080

